# Supplementary material for: Toward a Population-Based Breast Cancer Risk Stratification Approach? The Needs and Concerns of Healthcare Providers
Source: J Pers Med. 2021 Jun 10;11(6):540. doi: 10.3390/jpm11060540 (PMC8228184; doi:10.3390/jpm11060540)
Supplement: Supplementary file 1 [file jpm-11-00540-s001.zip › jpm-1228080-supplementary.pdf]

Table S1. Hierarchical tree of nodes along with their respective definitions, the number of respondents and additional remarks

LOGICAL MODEL COMPONENTS (meta-themes or dimensions)<br>Themes<br>**Nodes'** and sub-nodes' **names**	Definition of nodes	Nb of sources	Additional remarks
TARGETED POPULATION (WHO?)		10	
Eligible women	Aspects related to the targeted population: who should be eligible to the risk stratification services?	10	<u>Ambiguous</u><br>Respondents were not specifically questioned about the targeted population
• **Screening all women**	Opinions related to the inclusion of all women in the risk stratification approach	**4**	Concerns about feasibility and equity
• **Compared with the PQDCS**	Comparisons made with the current BC screening program (PQDCS) regarding the targeted population of a risk stratification approach	**9**	Difficult to replicate the model and should be complementary
CLINICAL ACTIVITIES (HOW? & WHAT?)	Aspects related to the clinical process steps of a future risk stratification program	15	(See each activity)
Identification & invitation	Strategies and ideas related to selection and invitation of women to the risk stratification approach	14	<u>Ambiguous</u><br>Closely intertwined with the targeted population
• **Identification or preselection**<br>• Referrals<br>• Selected risk factors<br>• Patients' self-reported questionnaire<br>• Through the PQDCS	Ways of preselecting women or improving referrals to specialized settings not to include all women in the risk stratification approach	**12**<br>7<br>6<br>6<br>4	Options should be evaluated in order to select a part of the population for feasibility, but there are important concerns about equity of access to services
• **Means of invitation**<br>• Letter (as the PQDCS)<br>• Via the physicians<br>• Social networks and family relatives	Means through which women could be invited to the risk stratification approach	**10**<br>6<br>7<br>2	Link to identification and preselection; concerns about feasibility and equity; no particular means of invitations were identified by respondents

**Risk assessment**	Aspects related to the current and expected tools or strategies used or to be used to assess BC risk	**15**	Activity discussed the most by respondents
• **Risk assessment tools (RATs)**<br>• Questionnaire and family history taking<br>• Risk prediction models (RPMs)<br>• Genetic tests	Types of tools currently used/known to be used or wanting to be developed to support HPs in terms of risk assessment	**15**<br>15<br>14<br>7	Concerns about the variety of each type of tool, the disparity of their use and their complexity (for GPs at least)
• **Assets of RPMs**<br>• Ease of use<br>• Clinical utility<br>• Low time or resources consuming	Expected qualities and characteristics of RPM to ensure their uptake and adoption by providers	**14**<br>9<br>14<br>4	Concerns about the usefulness of the tools to be proposed
**Risk communication**	Aspects related to the current and expected tools or strategies used or to be used to communicate BC risk	**15**	
• **Risk communication tools (RCTs)**<br>• Verbal explanations<br>• Visual (or graphical) representations<br>• Written information	Types of tools currently used/known to be used or wanting to be developed to support HPs' clinical practices in terms of risk communication	**15**<br>13<br>10<br>11	Concerns about the lack of RCTs and their diversity
• **Expected assets of RCTs**<br>• Adaptability or personalization to patients' risk<br>• Accessibility to patients<br>• Foster & facilitate exchange between professionals<br>• Ease of understanding	Expected qualities and characteristics of RCT to ensure their uptake and adoption by providers	**14**<br>13<br>9<br>6<br>5	
**Risk management**	Aspects related to current and expected tools or strategies used or to be used to managed BC risk	**15**	Should be sustained by tools developed to assess BC risk
• **Choices of recommendations**<br>• Patients' desires or choices<br>• Guidelines<br>• Patients' risk level<br>• Experience and clinical judgment<br>• Colleagues' expertise	Strategies and types of tools currently used/known to be used to support HPs' clinical practices in terms of risk management recommendations	**14**<br>9<br>9<br>8<br>7<br>7	Concerns about disparity of the recommendations (guidelines) among providers and health organizations that can lead to inequity in terms of management and follow-up
• **Follow-up**		10	

CONTRIBUTING FACTORS (WHICH?)	Factors that might affect the success of the BC risk stratification approach	15	
**Ethical principles**	Public healthcare leading values and ethical principles to respect for the services to be suitable and acceptable	**14**	
• Equity - Access to care		12	Link to [lack of] resources
• Precaution/non-maleficence		5	
• Utility and efficiency		5	
• Confidentiality (personal data)		2	
**Program organization**	Concerns about organizational process of the BC risk stratification approach	**13**	
• Feasibility		9	
• Complementarity with other services or programs		7	
• Development & management		6	
**Knowledge management**	Aspects related to the knowledge management tactics to be used within and surrounding the BC risk stratification approach	**15**	
• MDs' knowledge		15	
• Level of knowledge		13	
• Sources of information		15	
• Knowledge transfer tools		11	
• Diffusion of the strategy		9	
• HPs' sensitization		4	
• Public & patients' awareness		8	
• Available evidence-based knowledge		5	
**Human resources administration**	Opinions related to the current human resource management and challenges to overcome for the BC risk stratification approach to be acceptable and feasible	**14**	
• Current lack of human resources		12	
• HPs' workload and time		10	
• Limited resources in genetics		8	
• HPs' attitudes, beliefs or willingness		5	
• Roles and responsibilities		13	Discrepancies between type of HPs
• First line physicians		10	Concerns about the changes in their roles and responsibilities; doubts on its feasibility with the current resources
• Genetic specialists		5	
• Nurses & others allied HPs		5	

**ANTICIPATED RESULTS AND EFFECTS (WHY?)**	Expected effects or impacts of the implementation of a BC risk stratification set of services	**15**	
**Effects on patients or population**	At the individual level	**11**	
• Inducing negative psychological impacts		9	Link to the ethical principle of non-maleficence
• Increasing awareness and empowerment		5	
• Improving reassurance		3	
• Improving compliance		2	
**Effects on services**	At the organizational level	**15**	
• Improving quality of services		13	More accurate and personalized services
• Increasing demands for screening or assessment		8	Associated with increasing costs and pressures on HPs and organizations
• Standardizing and regulating practices		7	Seen as a means to reduce inequity between patients
• Supporting HPs' clinical practices		6	

Table S2. Examples of quotes codification according to each theme, by component of the logical model

Logical model components	*Themes* ∣*subthemes*∣	*[no of quotes indicated in table 3]* *Example of quotes* **(assigned respondents number)**
WHO? Target population	**Eligible respondents** ∣Comparisons with the PQDCS∣	*[Q1] "Currently, authorities cannot know all women's risk factors[…] it would be very time-consuming to estimate risk for each woman and then send them a letter recommending a tailored risk management strategy. It is impossible to imagine."* (R10)<br>*[Q2] "… So, I think it would be very difficult- if it is not quasi impossible- to do this as the PQDCS…"* (R16)
HOW? Clinical activities - WHAT? Associated strategies or tools	**Identification-Invitation** ∣Selection on the basis of RF∣	*[Q3] "I think that if we target women on the basis of high breast density or a positive family history, it's going to be easier, and maybe, we will be able to use the risk prediction model…"* (R8)<br>*[Q4] "For at-risk patients, without symptoms, I think we can use age, breast density and family history. With these three risk factors, I have the feeling that we get something here…"* (R13)
	**Risk assessment** ∣Clinical questionnaire∣	*[Q5] "The questionnaire is important; a detailed questionnaire… And sometimes, it is somewhat overlooked… so we have to go further with the patient [who has been referred]."* (R1)<br>*[Q6]"In my office, as a family physician, we use the personal breast and medical history in general - which may include chest radiation, as well as family history… Otherwise, at the breast clinic, we have more questions, like age at first birth, age at menarche…"* (R8)
	**Risk communication** ∣Lack of tool∣<br><br>∣Personalized RC∣	*[Q7] "We have some [tools] about screening or for those who have cancer. But for at-risk women, we have nothing like a leaflet that supports explanations or that vulgarizes the information."* (R12)<br>*[Q8] "[How risk is communicated] varies according to the patients. We have to adapt, and this is why I think we must have more than one tool to provide explanations about risk."* (R3)
	**Risk management** ∣(Lack of) Guidelines∣	*[Q9] "In my practice, the question that we have been asking the most is whether: 'when do we have to request another radiological modality than a mammogram in terms of follow-up?'[…] Up to now, I do not have much guidance. To my knowledge, there is not a lot of scientific evidence to guide us. It comes back to our experience and practice."* (R10)<br>*[Q10] "…[screening] is a continuous fight between oncologists and radiologists, even for standard modalities. Suppose a patient with a mother diagnosed with BC at 25. A common rule is to say 10 years minus the age, but we certainly do not begin at 15. So, do we have to start at 25? According to radiologists: no mammogram before 30. So, do we have to make an ultrasound, an MRI? I tell you, this zone is catastrophic."* (R13)

WHICH? Contributing factors	**Ethics**<br>❘Equity - access to care❘	*[Q11] "… the government does not pay for mutation on PALB2 because it is not considered as a high-risk gene. So, there are at-risk women for whom we do nothing. And for me, this is a problem because it is recognized as a BC risk and there are available interventions. But who decides when the risk is sufficiently high to do something?!"* (R17)<br>*[Q12] "Ideally, to be rational, it is women with cancer that should get access to an annual MRI in priority, and not her sister. Given that we have very limited resources, we have to target who can get access to services. You know that we do not really need a second line; we need a second line to decide who deserves to receive the services."* (R13)<br>*[Q13] "Doctors often do not have the resources to make the tests. I am hospital-based, and if I ask for a breast MRI, I have even more chances than a doctor in his private office […] If I ask it in 6 months, my patient will receive it in one year and a half, and I am in the hospital. So, imagine for those who are outside. Anyhow, it depends on the resources and the regions, but there are difficulties accessing radiological modalities."* (R12)
	**Program organization**<br>❘Complementarity with the PQDCS❘	*[Q14] "As I told you, there is a national committee who is working on when we should do a mammogram, an MRI or so on […] But it will not be complete because there is nothing about risk stratification, the genes or the genetics […] if you develop new tools that comprise the use of mammograms [and radiological modalities], it must be congruent with the recommendations of the national committee. Otherwise, we will be confused."* (R6)
	**Human resources administration**<br>❘Lack of resources❘	*[Q15] "Where I think there is a weakness, and this is not criticism: it is the lack of resources. When we refer to [name of BC centre] for managing a patient or doing a BRCA evaluation, they cannot. They have so many demands and waiting time; they experience staff shortage."* (R1)
	❘Roles and responsibilities❘	*[Q16] "I do a lot of personal risk assessment for physicians who asked for and found difficult to answer patients' inquiry [about BC risk]. But, is this my job? No, it is not my job to estimate BC risk for women of the general population [laughs]. My job is to take care of high-risk women."* (R16)<br>*[Q17] "This is more the domain of medical genetics. They could guide women when they find a mutation and they provide recommendations to attending physicians."* (R10)
	**Knowledge management**<br>❘MDs' knowledge❘	*[Q18] "[Genetic knowledge] should be developed. You know, it was restricted for a long time. Physicians who wanted to make tests, not fancy things, just BRCA for example, couldn't. It always needed the geneticist to adjudicate, instead of considering a genetic test as another normal clinical test. There are some efforts to do on this side, efforts of information and of vulgarization in order that physicians understand and [genetics] were no longer mysterious." (R9)*
	❘Knowledge transfer❘	*[Q19] "We must have good computerized medical records and the same for everybody, as well as governmental tools we can access in them. It should be integrated in our electronic system in which there is a tab for risk assessment. Once you have filled it up, it adds to the patient's medical records. It would be ideal…." (Respondent 10)*

	ǀAvailable evidence-base knowledgeǀ	*[Q20] "I would really like to have clear provincial recommendations so we can do all the same things. It upsets me when a woman gets a different healthcare plan or follow-up if she is seen at one centre instead of another. I think it is not fair and it is complicating things within families. Some patients live in a suburban area while some of their relatives downtown, and it is difficult to see that they don't get access to the same options..."* (R16)
WHY? Potential effects	**On services**	*[Q21] "I think it will allow targeting women who really need early screening. This will be the positive impact."* (R11)<br>*[Q22] "It would be useful to have criteria for Québec, to become more homogeneous and to streamline [practices]… We would feel more comfortable if everybody is doing the same thing. And if we have all the same tools and the same discourse, it will be more equal for women anywhere in Québec."* (R14)
	**On patients**	*[Q23] "Anxiety comes first to me. It is often the most anxious people who consulted for this kind of thing."* (R3)<br>*[Q24] "…closer investigations will lead to examine benign conditions and will contribute to increase patients' anxiety, that is clear."* (R13)
